# The Position of the Factory Nurse

**Published:** 1919-04-19

**Authors:** C. U. Kerr


### THE EDITOR'S LETTER-BOX.

### THE POSITION OF THE FACTORY NURSE.

*To the Editor of* The Hospital.

Sir,—"Shell Factory Nurse" must bear in mind that, as I have already pointed out, the Ambulance, or Nursing, Department in the factory, important as it is, is but *one* branch of industrial welfare. The employer chooses a superintendent with a general knowledge of industrial conditions and puts her in as "chief" of that department—to co-ordinate and direct such branches as (1) Employment, (2) Social Conditions, (3) Education, (4) Nursing, (5) Canteen, (6) Discipline, &c. The factory nurse and her department comes into being with the welfare department, and however much she may cavil at this arrangement she is subordinate to the welfare manager in the factory, just as the matron is her superior officer in the hospital. It would, therefore, be obviously absurd to give the factory nurse precedence over the welfare officer. You might just as well say she should be paid on an equal scale with the other members of the management of the firm. "Shell Factory Nurse" forgets that welfare superintendents are part of the management and responsible for many things besides the running of an ambulance department. Therefore, I think it is no disparagement to call it a step "up" when the nurse succeeds to the position of welfare superintendent, and adds to her duties and responsibilities, after having shown that she has the ability to grasp problems connected with the running of the factory.

Your correspondent is incorrect when she says "you will also note that the only promotion offered to the nurse is to be an assistant welfare supervisor." That is the first step—afterwards it stands to reason the nurse can go forward, if capable, to a post as chief superintendent. But to argue that a knowledge of nursing *alone* is adequate for running a welfare department is futile and a waste of time. If the factory nurse objects to working under the welfare officer she should seek a job in some other branch of nursing. But there, again, unless appointed as matron she would still not be supreme in authority.

In the words of Professor Urwick, the welfare superintendent must "be interested not in a single thing, physiological, physical, or psychical, but all combined. The position of the functionary having charge of the welfare department may, perhaps, be compared to that of the Dean in a really well-managed college, who appears to have no special work to do except sit in his room all day and receive visitors. Yet it is through his being there that the life of the college goes on, not only without friction but with real verve. The welfare worker in modern industry is the very antithesis of a legal officer."

The Welfare Workers' Association certainly does *not* attempt to define the salary, status and promotion of the factory nurse—such matters can only be decided by nurses themselves—but, on the other hand, the latter must remember that upon appointment they do not as a rule jump straight into office as head welfare superintendent without having had, *in addition to nursing*, the necessary instruction for such a position.—I am, Yours faithfully,

C. U. Kerr, LL.A. (Hons.).

